# Prevalence of left iliac vein compression on computed tomography scans from a population

**DOI:** 10.1590/1677-5449.190060

**Published:** 2020-08-31

**Authors:** Mateus Picada Corrêa, Guilherme Soldatelli Kurtz, Larissa Bianchini, Lauren Copatti, Marcelo Ribeiro, Jaber Nashat Saleh, Rafael Stevan Noel, Julio Cesar Bajerski

**Affiliations:** 1 Instituto Vascular – INVASC, Passo Fundo, RS, Brazil.; 2 Clínica Kozma, Passo Fundo, RS, Brazil.

**Keywords:** venous insufficiency, May-Thurner syndrome, varicose veins

## Abstract

**Background:**

May-Thurner syndrome (MTS) is defined as compression of the left iliac vein between the right iliac artery and the lumbar vertebral body in the presence of signs and symptoms of unilateral left chronic venous insufficiency. However, imaging findings of compression are not manifest in symptoms of the syndrome in all subjects.

**Objectives:**

To evaluate findings of compression in an asymptomatic population.

**Methods:**

Computed tomography angiographies or venous phase computed tomographies were analyzed. Demographic data and reason for the exam were recorded. Vein diameter was measured at the site of greatest compression and distal of the compression and the ratio between the two diameters was calculated.

**Results:**

From January to July of 2016, 590 computed tomography scans were analyzed (357 women and 233 men). Left iliac compression was found in 14.74% of patients. Patients with a left iliac diameter below the 5mm threshold had a mean diameter at the site of greatest iliac vein compression of 4.4 mm (range: 2.67 mm-4.97 mm). The ratio between the two measurements was < 0.5 in 30% of patients.

**Conclusions:**

Our study suggests that iliac vein compression is common among random patients who have had computed tomography for any other reason. This indicates that compression found on tomography images is not the only finding to consider when treating a patient.

## INTRODUCTION

May-Thurner Syndrome (MTS) is defined as compression of the left iliac vein (LIV) between the right iliac artery (RIA) and lumbar vertebral bodies in the presence of unilateral chronic venous hypertension involving the left lower limb (LLL). Studies indicate that MTS has greatest incidence among middle-aged women.^1^ The incidence in the general population of compression of the LIV by the RIA is not fully known. The objective of this study is to establish the prevalence of LIV compression in a population that is asymptomatic from a vascular point of view.

## METHODS

This study was authorized by the Ethics Committee at the Universidade de Passo Fundo, under registration number 98041418.5.0000.5342. It is a descriptive, cross-sectional, retrospective study. Images were analyzed from abdominal computed tomographies (CTs) with contrast and portal phase and from computed tomography angiographies that had been requested for the most varied range of reasons and were conducted by a private radiology and diagnostic imaging clinic (Clínica Kozma). This clinic has nine centers in the South of Brazil (Passo Fundo [two units], Erechim, Lagoa Vermelho, and Frederico Westphalen in the state of Rio Grande do Sul; Florianópolis, Chapecó, and Balneário Camboriú in the state of Santa Catarina; and Pato Branco in the state of Paraná). All images are stored on the same server.

**Figure 1:**
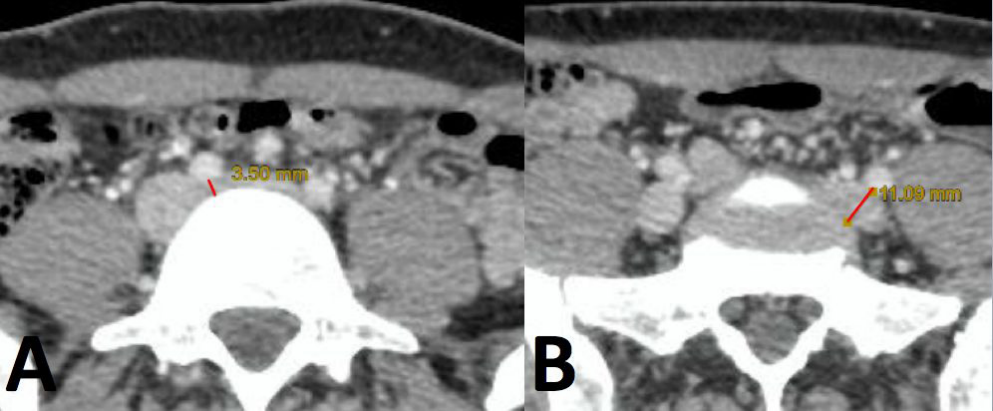
Diameters measured between the red lines. The point of greatest compression (A) and the last point of contact between the iliac vein and the vertebral body (B). This point was chosen because it is easy to reproduce.

The images were analyzed by two independent authors using image analysis software accessed via the internet (Animati Viewer^®^, Santa Maria, RS, Brazil). The diameter of the LIV was measured at its point of greatest compression and also in the first image in which it appears in contact with the vertebral body (Figure 1). Left iliac vein compression was defined as a diameter at the point of greatest compression of less than 5 mm. Examinations were excluded from the analysis if they did not include venous or portal phases, if LIV compression was secondary to other causes, such as tumors, or if the patient was known to have venous thrombosis of the iliofemoral axis. Patient characteristics were recorded including sex, age, and reason for requesting CT.

## RESULTS

From January 2016 to July 2016, 1,676 CTs were performed, 590 of which were candidates for the study. Of these, 357 were performed on women and 233 on men. The mean of age of patients was 53 years (range: 17-82).

Compression of the LIV was observed in 87 (14.74%) patients; 74 (85.05%) women and 13 (14.9%) men. The mean age of patients with compression was 41.4 years. Mean diameter was 4.4 mm at the point of compression for those patients whose LIV diameter was less than 5 mm, with a range of 2.67 mm to 4.97 mm. In the same subset of patients, mean LIV diameter in the first image in which it appears against the vertebral body was 11 mm. In 179 (30.3%) patients, the diameter at the point of greatest compression was no more than half of the diameter in the last image in which it appears against the vertebral body (ratio of 0.5).

In the subset of patients with compression (LIV < 5 mm), the reason for ordering the examination was known in 83 (46%) patients (Table 1). In 44 (53%) patients the reason was abdominal pains, 17 (20.4%) were examined to asses the urinary system, 12 (14.4%) were examined as part of onco-hematological follow-up, seven (8.4%) were asymptomatic, one (1.2%) was examined after polytrauma, one (1.2%) to assess toxoplasmosis, and one (1.2%) because of edema of the lower limbs. Only this last case was examined because of a reason that was possibly related with venous insufficiency, although the complaint was nonspecific.

**Table 1:** Reasons for ordering abdominal tomography in patients with LIV compression.

**Cause**	**n (%)**
Unexplained abdominal pains	44 (50)
Assessment of the urinary system	17 (19.3)
Oncological follow-up	12 (13.6)
Asymptomatic	7 (7.95)
Unknown	5 (5.6)
Polytrauma	1 (1.1)
Investigation of toxoplasmosis	1 (1.1)
Lower limb edema	1 (1.1)

n: number of patients.

## DISCUSSION

May-Thurner Syndrome comprises extrinsic compression of the left common iliac vein (LCIV) against bony structures by elements of the arterial system, accompanied by clinical symptoms of venous insufficiency.

Obstruction of the LCIV because of intraluminal adhesion was first described by McMurrich in 1906. Later, in 1957, May and Thurner detected lesions with fibrosis in this vessel in 22% of a series of 430 autopsies of cadavers. The clinical correlation was not reported until 1965, by Cockett and Thomas.^1^^,^^2^ Greater than 50% compression of the common iliac vein generally occurs against the lower lumbar vertebrae and should therefore be suspected in patients with scoliosis and dilated perimedullary veins.^3^ Both the pulsation and the chronic mechanical compression can cause intimal hypertrophy of the wall of the vessel, in addition to causing networks, channels, and deposits of collagen and intraluminal fibrin and, consequently, reduced distal flow and a resultant vascular gradient. This process involves two of the components of Virchow’s triad (endothelial damage and abnormal blood flow), which explains the predisposition to development of deep venous thrombosis (DVT).^4^

The exact prevalence of MTS has not been established, but it is estimated to vary in the range of 2-24% of people with some type of disorder of the venous system of the lower limbs.^5^ It is more common among women aged 18 to 50 years. Asymptomatic patients may have intravascular LIV injuries – detected by intravascular ultrasound – similar to those seen in patients with chronic venous insufficiency which, possibly, may be associated with future development of MTS.^6^ In other words, compression of the iliac vein may be asymptomatic until onset of the syndrome is triggered by an event such as gestation, prolonged immobilization, or surgery.

The clinical presentation of MTS includes persistent edema of the lower limb, with or without signs of venous hypertension. The reported prevalence of MTS in patients with venous thrombosis of the LLL varies in the range of 18-49%. Narayan et al. described a possible association between DVT and presence of > 70% stenosis of the LCIV.^7^ Carr et al. found a mean LCIV diameter of 6.5 mm in the general population and 4 mm in patients with DVT, demonstrating a six-times greater risk of DVT in patients with a 4mm diameter.^8^ Each 1 millimeter reduction in LCIV diameter increased the likelihood of DVT by a factor of 1.68.

Different patterns of iliac vein compression have been described in patients with chronic venous disease. Compression by the right common iliac artery remains the most common (77.5%), followed by a combination of the right and left common iliac arteries (47.5%), and then compression by the left common iliac artery only (18%).^9^^,^^10^

Venous phase CT images enable direct visualization of the action of venous compression, of cases of thrombosis, and of collateral circulation.^11^^,^^12^ The advantages of CT in relation to Doppler ultrasound include shorter duration, not being examiner-dependent, and a better view of the pelvic veins. However, CTs require large volumes of contrast and cannot be used during pregnancy or in people with renal dysfunction.^2^ Additionally, there is variability in diameter measurements that is dependent on respiratory phasicity and dorsal decubitus. When compared to digital subtraction phlebography (DSP), the cross-sectional diameter and area measured on CT correlate with reflux shown on DSP.^13^ Another effective imaging method is intravascular ultrasound (IVUS), which, in addition to determination of the degree of stenosis, is useful for calibrating the vessel before deployment of the stent.^14^ Notwithstanding, these last two methods both involve the inconvenience of invasivity.

Kibbe et at.^15^ assessed 50 patients seen in emergency for abdominal pains and observed that 24% of them had > 50% LCIV compression, and 66% had > 25% compression. Mean compression was 35.5% (5.6-74.8%) and in 84% of cases it was caused by the right common iliac artery.

Recently, a Chinese study using CTs assessed the incidence of LCIV compression in 500 asymptomatic patients and found stenosis exceeding 25% and 50% in 37.8% and 9.8% of those analyzed, respectively. After 39.5 months’ follow-up, the incidence of MTS was 1.6% of patients who had originally been asymptomatic. Additionally, the degree of stenosis was an independent risk factor for development of MTS (Wald chi-square = 8.84, hazard ratio = 1.13, p < 0.001).^16^

Tomographic studies conducted with 10 patients who had LCIV compression observed a mean diameter at the origin of the LCIV of 3.5 mm (1-8.5 mm), whereas in the control group the equivalent diameter was 11.5 mm (6.3-16.1 mm) (p < 0.01). The mean percentage LCIV stenosis due to compression by the right common iliac artery was 68%.^17^

Nazzal et al.^18^ reported that when they compared the LIV diameter at the point of greatest compression with the diameter of the segment distal to the compression, the rate of compression was 36.6% in the male subset of the population they studied and 48.5% in the female subset. Additionally, there was > 70% LCIV compression in 30.6% of 300 patients analyzed, once more disproportionately more prevalent in the females (19.5% vs. 11.1%, p < 0.049).

Ou-Yang et al.^19^ categorized patients with iliac vein compression into three groups – those with simple MTS, those with MTS related to lumbar degeneration, and those with MTS due to other causes – and reported that the type of MTS has a direct relationship with treatment results (Wald chi-square = 6.092, p = 0.009). They reported a cutoff point of 2.98 mm for MTS, with diagnostic sensitivity of 90% and 100% specificity.

Certain details should be made clear with regard to our study. The study period was chosen merely as a means of limiting the sample size and was chosen at random. When planning the study, we found that there is no agreement in the literature on measurement of stenosis in LIV. Whether at the site of analysis or at the cutoff point for cross-sectional diameter that determines compression, each author uses a different measurement. The choice of the point of greatest compression is very often subjective, because the artery may follow an oblique path across the vein and so compression of the anterior wall may not be uniform. In view of this, we decided to conduct measurements using a ratio, choosing an easily-identified point upstream: the point at which the common iliac vein touches the vertebral body. If venous hypertension is present, there is a greater possibility of venous dilation occurring proximal to the left hypogastric vein, which would act as the hypertension drainage vessel, facilitating diagnosis.

Use of software for manipulation of tomographic images facilitates diagnosis of venous compression, but it is not available at all centers and expertise is needed to achieve an effective reconstruction. In our study, we analyzed the raw axial data using freeware cloud-based software, with the objective of mimicking any software for analysis of axial images, suggesting universalization of the methodology.

Our study suggests that compression of the LIV is common on CTs from random patients with no knowledge of the existence of chronic venous insufficiency or DVT in LLL. However, the study has a limitation. Since the examinations were conducted in private imaging clinics, which are not all located in hospitals, we did not have access to clinical data on all of the patients, which were only available for examinations conducted in hospitals where the clinic has a branch or when the treating physician specified the clinical indications when requesting the examination. We believe that a study in which the reason the examination was ordered was available for all examinations analyzed would carry much more weight. Nonetheless, the results confirm, and are similar to, data already available in the literature and add weight to the conclusion that compression does not necessarily result in MTS. Consequently, only compressions associated with symptoms should be treated.

## CONCLUSIONS

This study suggests that compression of the LIV is common on CTs from random patients with no knowledge of the existence of chronic venous insufficiency or DVT in LLL, confirming published data and showing a higher prevalence among females.

In view of the results of this study, we suggest as an objective for future studies the establishment of correlations between the LIV diameter at its point of greatest compression and its relationship with the vein upstream as a feasible tool for assessment of the presence of compression, in addition to analysis of venous hypertension upstream, with presence of pelvic varicose veins originating from the left hypogastric vein.
