# Supplementary material for: Prevalence of left iliac vein compression on computed tomography scans from a population
Source: J Vasc Bras. 2020 Aug 31;19:e20190060. doi: 10.1590/1677-5449.190060 (PMC8202169; doi:10.1590/1677-5449.190060)
Supplement: Translation

# Translation: Prevalência de compressão da veia ilíaca esquerda em imagens tomográficas de uma população

**Authors:** Mateus Picada Corrêa, Guilherme Soldatelli Kurtz, Larissa Bianchini, Lauren Copatti, Marcelo Ribeiro, Jaber Nashat Saleh, Rafael Stevan Noel, Julio Cesar Bajerski

**Affiliations:** 1 Instituto Vascular – INVASC, Passo Fundo, RS, Brasil.; 2 Clínica Kozma, Passo Fundo, RS, Brasil.

**Keywords:** insuficiência venosa, síndrome de May-Thurner, varizes

## Abstract

**Contexto:**

A síndrome de May-Thurner (SMT) é a compressão da veia ilíaca esquerda (VIE) entre a artéria ilíaca direita e o corpo vertebral associada à hipertensão venosa crônica unilateral no membro inferior esquerdo. Porém, o achado tomográfico da compressão não necessariamente se reflete em sintomas.

**Objetivos:**

Avaliar o achado de compressão da veia ilíaca esquerda em tomografias realizadas por outros motivos.

**Métodos:**

Angiotomografias ou tomografias computadorizadas (TCs) com fase venosa foram analisadas. Foram coletados os dados demográficos e o motivo do exame, quando presente, e foi analisada a relação do diâmetro da veia ilíaca esquerda no ponto de maior compressão com um ponto a montante.

**Resultados:**

De janeiro a julho de 2016, 590 tomografias foram analisadas, sendo 357 de mulheres e 233 de homens. A compressão da VIE ocorreu em 87 (14,74%) pacientes, dos quais 74 (85,05%) eram mulheres e 13 (14,9%) homens. O diâmetro médio do ponto de maior compressão entre os pacientes que apresentavam VIE < 5 mm foi de 4,4 mm, variando de 2,67 mm a 4,97 mm. O diâmetro no ponto de maior compressão representou até metade do diâmetro na última imagem justaposta ao corpo vertebral (índice de 0,5) em 179 (30,3%) dos pacientes.

**Conclusões:**

Nosso estudo sugere que a ocorrência de compressão da VIE em TC de pacientes aleatórios, sem conhecimento de insuficiência venosa crônica ou TVP em MIE, é comum. Isso mostra que o achado tomográfico de compressão não necessariamente resulta em sintomas e não deve ser a única razão para tratar um paciente.

## INTRODUÇÃO

A síndrome de May-Thurner (SMT) é definida como a compressão da veia ilíaca esquerda (VIE) entre a artéria ilíaca direita (AID) e o corpo das vértebras lombares associada à hipertensão venosa crônica unilateral no membro inferior esquerdo (MIE). Os estudos apontam um predomínio da SMT em mulheres de meia-idade^1^. A incidência da compressão da VIE pela AID na população em geral não é propriamente conhecida. Este estudo visa estabelecer a prevalência de compressão da VIE em uma população assintomática do ponto de vista vascular.

## MÉTODOS

O presente estudo foi autorizado pelo Comitê de Ética da Universidade de Passo Fundo, pelo código 98041418.5.0000.5342. É um estudo retrospectivo, descritivo, do tipo transversal. Foram analisadas as imagens de angiotomografias computadorizadas e tomografias computadorizadas (TCs) abdominais com contraste que continham fase portal realizadas de forma consecutiva pelos mais diversos motivos em uma clínica privada de radiologia e diagnóstico por imagem (Clínica Kozma). Essa clínica apresenta nove centros no sul do Brasil [Passo Fundo (duas unidades), Erechim, Lagoa Vermelha e Frederico Westphalen no Rio Grande do Sul; Florianópolis, Chapecó e Balneário Camboriú em Santa Catarina; e Pato Branco no Paraná], e todas as imagens dos exames são armazenadas em um servidor comum.

Os exames foram analisados por dois autores independentes, em um *software* de análise de imagens com acesso pela internet (Animati Viewer^®^, Santa Maria, RS, Brasil). Foram avaliados os diâmetros da VIE em seu ponto de maior compressão e o diâmetro da VIE em sua primeira imagem justaposta ao corpo vertebral (Figura 1). Foi considerada compressão da VIE quando seu diâmetro no ponto de maior compressão foi menor que 5 mm. Foram excluídos da análise exames que não apresentavam fase venosa ou portal, exames em que a compressão da VIE era originada de outras causas, como compressões neoplásicas, ou exames de pacientes sabidamente com trombose venosa de eixo ilíaco-femoral. Foram descritas as características dos pacientes como: sexo, idade e motivo da realização da TC.

**Figura 1:** Locais de avaliação dos diâmetros entre as linhas vermelhas. O local de maior compressão (A) e o último local de contato da veia ilíaca com o corpo vertebral (B). Esse local foi escolhido por ser de fácil reprodução.

## Resultados

No período entre janeiro de 2016 e julho de 2016, 1.676 TCs foram realizadas. Destas, 590 exames eram candidatos ao estudo, entre os quais 357 foram realizados em mulheres e 233 de homens. A média de idade dos pacientes avaliados foi de 53 anos (17 até 82).

A compressão da VIE ocorreu em 87 (14,74%) pacientes, sendo que, destes, 74 (85,05%) eram mulheres e 13 (14,9%) homens. A idade média dos pacientes com compressão era de 41,4 anos. O diâmetro médio do ponto de maior compressão entre os pacientes que apresentavam VIE menor que 5 mm foi de 4,4 mm, variando de 2,67 mm a 4,97 mm. Já o diâmetro da VIE na primeira imagem justaposta ao corpo vertebral no mesmo grupo de pacientes teve como média 11 mm. O diâmetro no ponto de maior compressão representou até metade do diâmetro na última imagem justaposta ao corpo vertebral (índice de 0,5) em 179 (30,3%) dos pacientes.

No grupo de pacientes com compressão (VIE < 5 mm), o motivo da solicitação do exame era conhecido em 83 (46%) dos pacientes (Tabela 1). Em 44 destes (53%), o motivo foi dor abdominal, 17 (20,4%) realizaram o exame para avaliação do sistema urinário, 12 (14,4%) realizaram o exame em acompanhamento onco-hematológico, sete (8,4%) eram assintomáticos, um (1,2%) realizou o exame devido a politrauma, um (1,2%) o realizou para avaliar toxoplasmose, e um (1,2%) o realizou devido a edema de membros inferiores. Somente o último caso apresentou como motivo uma possível relação com insuficiência venosa, porém a queixa era inespecífica.

**Tabela 1:** Motivos de realização da tomografia abdominal nos pacientes portadores de compressão da VIE.

**Causa**	**n (%)**
Dor abdominal a esclarecer	44 (50)
Avaliação do sistema urinário	17 (19,3)
Acompanhamento oncológico	12 (13,6)
Assintomáticos	7 (7,95)
Desconhecido	5 (5,6)
Politraumatizado	1 (1,1)
Avaliação de toxoplasmose	1 (1,1)
Edema de membros inferiores	1 (1,1)

n: número de pacientes.

## DISCUSSÃO

A SMT consiste na compressão venosa extrínseca da veia ilíaca comum esquerda (VICE) contra estruturas ósseas pelo sistema arterial, acompanhada de sintomas clínicos de insuficiência venosa.

A obstrução da VICE devido a adesões intraluminais foi descrita primeiramente por McMurrich em 1906 e, em 1957, May e Thurner detectaram lesões fibrosas nesse vaso em 22% das autópsias de 430 cadáveres. A correlação clínica somente foi relatada por Cockett & Thomas em 1965^1^^,^^2^. A compressão da veia ilíaca comum > 50% geralmente ocorre contra as vértebras lombares inferiores e, por isso, deve ser suspeitada em pacientes com escoliose e veias perimedulares dilatadas^3^. Tanto a pulsação quanto a compressão mecânica crônicas podem resultar em hipertrofia intimal da parede do vaso, além de causar redes, canais e depósitos de colágeno e fibrina intraluminais e, consequentemente, diminuição do fluxo distal e geração de um gradiente vascular. Esse processo possui dois componentes da tríade de Virchow (dano endotelial e fluxo sanguíneo anormal), o que justifica a predisposição a desenvolver trombose venosa profunda (TVP)^4^.

A prevalência exata da SMT não é estabelecida, mas estima-se que varie de 2-24% dos indivíduos com algum distúrbio do sistema venoso dos membros inferiores^5^. É mais comum em mulheres, entre os 18 e os 50 anos de idade. Pacientes assintomáticos podem apresentar lesões intravasculares da VIE – detectadas pelo ultrassom intravascular – similares às de portadores de insuficiência venosa crônica que, possivelmente, se associam ao desenvolvimento futuro de SMT^6^. Ou seja, a compressão da veia ilíaca pode ser assintomática até que algum evento desencadeie o início da síndrome, como gestação, imobilização prolongada ou cirurgia.

O quadro clinico da SMT inclui edema persistente do membro inferior, com ou sem estigmas de hipertensão venosa. A prevalência reportada de SMT em pacientes com trombose venosa de MIE varia de 18-49%. Narayan et al. descreveram possível associação entre TVP e presença de estenose > 70% da VICE^7^. Carr et al. encontraram diâmetro médio da VICE de 6,5 mm na população geral e de 4 mm em pacientes com TVP, evidenciando-se risco seis vezes maior de TVP nos pacientes com diâmetro de 4 mm^8^. Nesse sentido, cada milímetro menor no diâmetro da VICE aumentou as chances de TVP por um fator de 1,68.

Diferentes padrões de compressão da veia ilíaca em pacientes com doença venosa crônica já foram descritos, permanecendo a compressão pela artéria ilíaca comum direita a anomalia mais comum (77,5%), seguida pela associação entre as artérias ilíacas comum direita e esquerda (47,5%) e compressão pela artéria ilíaca comum esquerda isoladamente (18%)^9^^,^^10^.

A imagem na fase venosa da TC permite a visualização direta da compressão venosa, de casos de trombose e da circulação colateral^11^^,^^12^. As vantagens da TC em relação ao Doppler incluem menor tempo de exame, não ser examinador-dependente e possuir melhor visualização das veias pélvicas. Contudo, a TC necessita de grande volume de contraste e não pode ser realizada durante a gravidez ou em indivíduos com alteração da função renal^2^. Além disso, possui variabilidade de diâmetro com a fasicidade respiratória e o decúbito dorsal. Quando comparada à flebografia por subtração digital (FSD), o diâmetro e a área transversal aferidos pela TC apresentam correlação com o refluxo presente na FSD^13^. Outro método de imagem efetivo é o ultrassom intravascular (IVUS), o qual, além de determinar o grau de estenose, é capaz de calibrar o vaso antes da liberação do stent^14^. Estes últimos possuem, contudo, o inconveniente de serem métodos invasivos.

Kibbe et al.^15^ avaliaram 50 pacientes atendidos na emergência devido a dor abdominal e observaram que 24% deles apresentavam compressão da VICE > 50%, e 66% possuíam compressão > 25%. A compressão média foi de 35,5% (5,6-74,8%), e em 84% dos casos foi causada pela artéria ilíaca comum direita.

Recentemente, um estudo chinês utilizando TC avaliou a incidência de compressão da VICE em 500 pacientes assintomáticos e encontrou estenose maior que 25% e 50% em 37,8% e 9,8% dos indivíduos, respectivamente. Em 39,5 meses de seguimento, a incidência de SMT foi de 1,6% dos pacientes originalmente assintomáticos. Além disso, o grau de estenose foi um fator de risco independente para o desenvolvimento de SMT (qui-quadrado de Wald = 8,84, *hazard ratio* = 1,13, p < 0,001)^16^.

Estudos tomográficos realizados em 10 pacientes que apresentavam compressão da VICE demonstraram diâmetro médio na origem da VICE de 3,5 mm (1-8,5 mm), enquanto que no do grupo controle esse diâmetro foi de 11,5 mm (6,3-16,1 mm) (p < 0,01). A porcentagem média da estenose de VICE devido a compressão pela artéria ilíaca comum direita foi de 68%^17^.

Nazzal et al.^18^ relataram que, quando comparado o diâmetro do ponto de maior compressão da VIE com o do segmento distal a ele, a taxa de compressão foi de 36,6% na população masculina estudada, e 48,5% na feminina. Ademais, houve compressão > 70% da VICE em 30,6% de 300 pacientes analisados, também predominantemente no sexo feminino (19,5% *versus* 11,1%, p < 0,049).

Ou-Yang et al.^19^ categorizaram os pacientes com compressão da veia ilíaca em três grupos – aqueles com SMT simples, SMT relacionada à degeneração lombar, e SMT por outras causas – e relataram que o tipo de SMT apresenta relação direta com o resultado terapêutico (qui-quadrado de Wald = 6,092, p = 0,009). Além disso, o ponto de corte para SMT foi 2,98 mm, com sensibilidade diagnóstica de 90% e especificidade de 100%.

Algumas considerações sobre nosso trabalho devem ser elencadas. A decisão de utilizar o período de tempo do estudo visava apenas limitar a amostra e foi aleatória. Evidenciamos, durante o planejamento deste estudo, que não há concordância na literatura da medida de estenose na VIE. Seja no sítio de análise ou no ponto de corte do diâmetro transverso que determina compressão, cada autor utiliza uma forma de medida. A escolha do ponto de maior compressão muitas vezes é subjetiva, pois a artéria pode ter um trajeto oblíquo sobre a veia, não comprimindo de forma homogênea a sua parede anterior. Por isso, optamos por realizar a medida por meio de um índice, escolhendo um ponto a montante de fácil identificação em qualquer análise: o local onde a veia ilíaca comum toca o corpo vertebral. Caso exista hipertensão venosa, há uma maior possibilidade de ocorrer dilatação venosa proximal à veia hipogástrica esquerda, que serviria como vaso de drenagem da hipertensão, facilitando o diagnóstico.

A utilização de *softwares* de manipulação de imagens tomográficas facilita o diagnóstico da compressão venosa; todavia, não estão disponíveis em todos os centros, além de necessitarem de *expertise* para uma reconstrução efetiva. No nosso estudo, analisamos os dados brutos axiais utilizando um *software freeware* da nuvem, com o objetivo de mimetizar qualquer *software* de análise de imagens axiais, sugerindo uma universalização da metodologia.

Nosso estudo sugere que a compressão da VIE em TC de pacientes aleatórios, sem conhecimento da existência de insuficiência venosa crônica ou TVP em MIE, é comum. Porém, o estudo possui uma limitação. Por ter sido realizado em clínicas particulares de imagem, nem sempre situadas em instituições hospitalares, não tivemos acesso aos quadros clínicos de todos os pacientes, encontrados apenas nos exames realizados em hospitais onde há uma filial da clínica ou quando havia indicação clínica na solicitação do exame pelo médico assistente. Acreditamos que um estudo em que todos os exames tenham o motivo da solicitação teria muito mais força. Mesmo assim, os resultados corroboram e são semelhantes aos já encontrados na literatura e reforçam a conclusão de que a compressão não resulta necessariamente em SMT. Consequentemente, somente compressões associadas a sintomas devem ser tratadas.

## CONCLUSÃO

O presente estudo sugere que a ocorrência de compressão da VIE em TC de pacientes aleatórios, sem conhecimento da existência de insuficiência venosa crônica ou TVP em MIE, é comum, corroborando os dados da literatura, com uma prevalência maior no sexo feminino.

De acordo com os resultados do estudo, sugerimos como alvo de novas pesquisas o estabelecimento de uma correlação do diâmetro no ponto de maior compressão da VIE e sua relação com a veia a montante como uma ferramenta viável de avaliação da presença da compressão, além da análise de hipertensão venosa a montante, com a presença de varizes pélvicas de origem em veia hipogástrica esquerda.
